# Genetic Polymorphism of* msp*1 and* msp*2 in* Plasmodium falciparum* Isolates from Côte d'Ivoire versus Gabon

**DOI:** 10.1155/2016/3074803

**Published:** 2016-03-24

**Authors:** William Yavo, Abibatou Konaté, Denise Patricia Mawili-Mboumba, Fulgence Kondo Kassi, Marie L. Tshibola Mbuyi, Etienne Kpongbo Angora, Eby I. Hervé Menan, Marielle K. Bouyou-Akotet

**Affiliations:** ^1^Malaria Research and Control Centre, National Institute of Public Health, BPV 47, Abidjan, Côte d'Ivoire; ^2^Faculty of Pharmacy, Department of Parasitology and Mycology, Félix Houphouët-Boigny University, BPV 34, Abidjan, Côte d'Ivoire; ^3^Faculty of Medicine, Department of Parasitology and Mycology, University des Sciences de la Santé, BP 4009, Libreville, Gabon; ^4^Parasitology and Mycology Laboratory of Diagnosis and Research Centre on AIDS and Other Infectious Diseases, 01 BPV 03, Abidjan, Côte d'Ivoire

## Abstract

*Introduction*. The characterization of genetic profile of* Plasmodium* isolates from different areas could help in better strategies for malaria elimination. This study aimed to compare* P. falciparum* diversity in two African countries.* Methods*. Isolates collected from 100 and 73* falciparum* malaria infections in sites of Côte d'Ivoire (West Africa) and Gabon (Central Africa), respectively, were analyzed by a nested PCR amplification of* msp*1 and* msp*2 genes.* Results*. The K1 allelic family was widespread in Côte d'Ivoire (64.6%) and in Gabon (56.6%). For* msp*2, the 3D7 alleles were more prevalent (>70% in both countries) compared to FC27 alleles. In Côte d'Ivoire, the frequencies of multiple infections with* msp*1 (45.1%) and* msp*2 (40.3%) were higher than those found for isolates from Gabon, that is, 30.2% with* msp*1 and 31.4% with* msp*2. The overall complexity of infection was 1.66 (SD = 0.79) in Côte d'Ivoire and 1.58 (SD = 0.83) in Gabon. It decreased with age in Côte d'Ivoire in contrast to Gabon.* Conclusion*. Differences observed in some allelic families and in complexity profile may suggest an impact of epidemiological facies as well as immunological response on genetic variability of* P. falciparum*.

## 1. Background

The World Health Organization (WHO) reported recently a significant decline in the global malaria burden over the last 15 years, achieving the 2000 Millennium Development Goals. Fifty-seven countries have reduced their malaria cases by 75%, in line with the World Health Assembly's target for 2015. Malaria mortality rates in Africa have fallen by 66% among all age groups and by 77% among children under 5. The progress made in reduction of malaria burden is attributed to massive rollout of effective prevention and treatment tools, including insecticide-treated nets and implementation of Artemisinin-based Combination Therapies (ACTs) as the first-line treatment [1].

Regardless of the progress made, Africa continues to shoulder the majority of malaria burden. Côte d'Ivoire and Gabon are two sub-Saharan Africa (SSA) countries with different malaria endemicity. In Côte d'Ivoire, located in West Africa, malaria accounts for 43% of all causes of outpatient visits. The disease is responsible for one-third of reported deaths in health facilities in the country [2]. In Gabon, a Central African country, malaria prevalence varied between 2005 and 2011. After a trend of a reduction between 2005 and 2008, a rebound of malaria cases prevalence was observed in the rural and urban areas [3]. Further, malaria risk shifted towards older children and adults who are thought to have acquired premunition [3, 4]. Some of the factors that hinder progress in malaria control and slow down the elimination agenda include the emergence and spread of drug-resistant parasite strains and development of vectors resistant to insecticide [5–7]. Development of a malaria vaccine would be an ideal addition to the existing tools being deployed towards malaria control and elimination. However, one of the limitations of the development of malaria vaccine is the extensive genetic diversity in parasite populations limiting the efficacy of acquired protective immunity to malaria [8]. Individuals are often simultaneously infected by multiple parasite clones that are related to the transmission intensity and are described as a factor determining the host immune status [9–11]. This situation may have an impact on the efficacy of malaria vaccine and clinical issue of the disease. It is therefore important to characterize the parasite populations in order to adapt malaria control and elimination strategies.

To be successful, malaria elimination will require knowledge of parasite genome variation in different geographical locations and a better understanding of the factors that determine gene flow between locations [12]. Merozoite surface protein-1 (msp-1) and merozoite surface protein-2 (msp-2) which are asexual blood stage antigens are considered as prime candidates for the development of a malaria vaccine and are also suitable markers for the identification of genetically distinct* Plasmodium falciparum* parasite populations [8, 11, 13].

There are a limited number of studies that have compared genetic profiles of malaria parasites from different African countries particularly by genotyping* msp*1 and* msp*2 genes. This study aimed to compare the genetic diversity of* P. falciparum* in infected patients living in two different areas, Côte d'Ivoire and Gabon, by using these two highly polymorphic genes [13–15].

## 2. Methods

### 2.1. Study Areas

This study was carried out in Côte d'Ivoire and in Gabon.

In Côte d'Ivoire, isolates have been collected in four of the six sentinel sites of malaria surveillance of the National Malaria Control Program (NMCP), namely, Abidjan, the economic capital city located in the south-east, Abengourou in the east, San Pedro in the southwest, and Yamoussoukro in the center of the country. Abidjan and San Pedro are coastal and forest areas with a hot and humid climate. Abengourou is a forest area with a subequatorial climate. Yamoussoukro is a forest transition zone with a tropical climate. In Côte d'Ivoire, malaria is endemic and predominantly caused by* Plasmodium falciparum* [2, 16]. The transmission is perennial with peaks during rainy seasons (from April to August and from October to November).

In Gabon, parasites were collected at the Centre Hospitalier Universitaire de Libreville (CHL) and the Regional Hospital of Melen (RHM), two sentinel sites for malaria surveys of the NMCP. The CHL is located in Libreville, the capital city, and the RHM is in a suburban area located 11 km north of Libreville. Malaria transmission, predominantly caused by* P. falciparum,* is perennial without significant fluctuation throughout the year in Gabon [3]. The climate is equatorial in the whole country.

### 2.2. Study Design and Patients Enrolment

Patients from Côte d'Ivoire were enrolled during a prospective randomized control survey assessing Artemisinin-based Combination Therapies (ACTs) according to the standard WHO 2003 efficacy assessment protocol conducted in 2012 at Abengourou, San Pedro, and Yamoussoukro [17] and in 2013-2014 at Abidjan (Yavo et al., nonpublished data).

In Gabon, prospective cross-sectional surveys were conducted at Libreville and Melen in 2011. In these sentinel sites, the study team collected data during at least one rainy and one dry season. In each site, febrile outpatients and inpatients are routinely screened for* P. falciparum* infection. Body temperature, history of fever, age, sex, bed net use, home treatment with antimalarial drug, and location were collected. Data on self-medication have been collected through a detailed CRF. Patients were asked about previous history of fever and drug intake the month before the consultation, the type of molecule taken, and the duration of the treatment during each survey throughout the study period [4].

Before inclusion, written informed consent was obtained from the patient or the patient's legal guardian (for children). Approvals were obtained from the national ethic committee in Côte d'Ivoire and the Ministry of Health in Gabon.

### 2.3. Parasite DNA Extraction

After malaria diagnostic tests, thick and thin blood smears and parasitized blood samples were collected on filter paper in both countries. DNA from* P. falciparum* isolates from Côte d'Ivoire was extracted using Chelex method as previously described [18]. Nucleic acids extraction from Gabon samples was performed using the QIAamp kit (QIAGEN®) according to the manufacturer's instructions.

### 2.4. *P. falciparum* Genotyping

For all* P. falciparum* isolates genotyping, the two polymorphic loci* msp*1 and* msp*2 were analyzed together by using nested PCR. Primary amplifications followed by secondary PCR reactions using specific primers for K1, Mad20, and RO33 (for* msp*1) and 3D7 and FC27 (for* msp*2) allelic families were performed. The PCR primers sequences and cycles were previously described [13]. All isolates have been amplified at the Diagnosis and Research Centre on AIDS and Other Infectious Diseases of Abidjan, Côte d'Ivoire, with the same reagents and bench. Each genotype was identified based on the size of the PCR products using a 1.5% agarose gel. A 0.5 *μ*g/mL of ethidium bromide was used for the UV detection. Electrophoresis conditions were 100 mV for 30 min.

Bands were visualized under an UV transilluminator (BIOCOM*™*) and fragment sizes estimated by comparison to the 1 kb plus DNA ladder (SmartLadder*™* small fragment, EUROGENTEC).

### 2.5. Definitions

The detection of a single PCR fragment for each locus was classified as an infection with one parasite genotype. The detection of more than one PCR fragment for either* msp*1 or* msp*2 loci (i.e., an infection with more than one parasite genotype) was defined as a multiple* P. falciparum *infection. The number of patients with more than one parasite genotype out of the number of infected population was defined as the frequency of multiple infections (MI). The complexity was defined as the mean number of parasite genotypes per infected patient [19].

### 2.6. Statistical Analysis

All data were recorded using Epi data version 3.1 and analyzed with SPSS for windows (version 16.0). The frequency of each allele was estimated over all the alleles detected. Allelic family distribution and the number of genotypes detected in each infected patient were calculated according to the site. The complexity of infection was determined according to the site and the age. The complexity of infection and the frequency of multiple infections were calculated by combining the* msp*1 and* msp*2 PCR genotyping results. The highest number of bands detected, whatever the locus, was used to calculate the value for the overall complexity of infection.

Differences between groups were assessed using Chi-square or Mann-Whitney tests. The level of significance for statistical tests was set at 0.05.

## 3. Results

A total of 173 parasite DNA samples were analyzed: 100 from Côte d'Ivoire and 73 from Gabon.

### 3.1. Characteristics of Both Study-Sites Patients

The median age of patients from Côte d'Ivoire was 11 years (min. = 1 year; max. = 74 years). It was 16 years for the patients from Gabon (min. = 1 year and max. = 80 years).

### 3.2. *msp*1 and* msp*2 Genes Genotyping

#### 3.2.1. Allelic Families Frequency

*msp*1 and* msp*2 genes have been successfully amplified in 82% (82/100) and 72% (72/100) samples from Côte d'Ivoire, respectively. Likewise, amplification rate was of 72.6% (53/73) for* msp*1 gene and 69.9% (51/73) for* msp*2 gene in isolates from Gabon.

The distribution of the different allelic families of* msp*1 and* msp*2 genes is shown in Table 1. In both areas, K1-type alleles and 3D7 type alleles were the most frequent. Allelic families' frequencies were not statistically different between areas from both countries.

#### 3.2.2. Allelic Diversity

**Figure 1:**
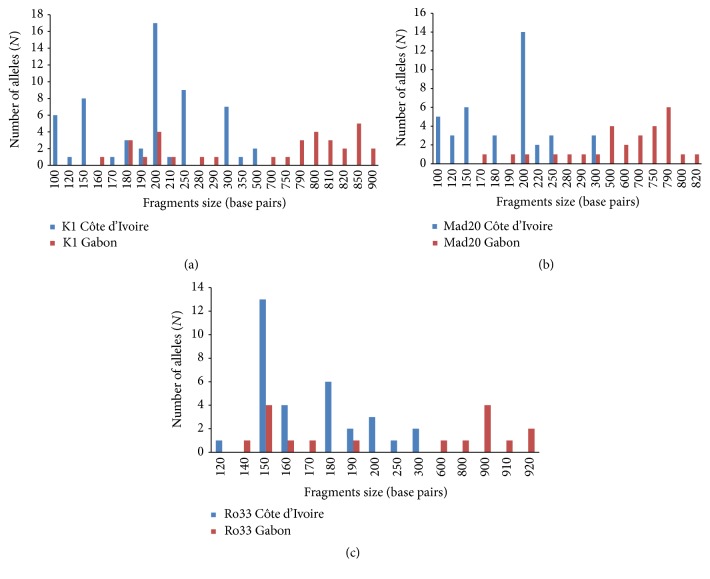
(a)* P. falciparum msp*1 K1 type alleles classified according to the length (in base pairs) in Côte d'Ivoire and Gabon. (b) * P. falciparum msp*1 Mad20 type alleles classified according to the length (in base pairs) in Côte d'Ivoire and Gabon. (c)* P. falciparum msp*1 RO33 type alleles classified according to the length (in base pairs) in Côte d'Ivoire and Gabon.

*msp*1 gene analysis showed that 12 K1 type alleles with a size ranging from 100 to 500 bp, 8 Mad20 type alleles (100–300 bp), and 8 RO33 type alleles (120–300 bp) were identified in* P. falciparum* isolates from Côte d'Ivoire. In Gabon, 15 K1 type alleles with a size ranging from 160 to 900 bp, 14 Mad20 type alleles (170–820 bp), and 10 RO33 type alleles (140–920 bp) were found. K1 200 bp, Mad 200 bp, and RO33 150 bp alleles were predominant in Côte d'Ivoire. In Gabon, K1 850 bp, Mad 790 bp, and RO33 (150 bp and 900 bp) alleles were the most frequent (Figures 1(a), 1(b), and 1(c)).

**Figure 2:**
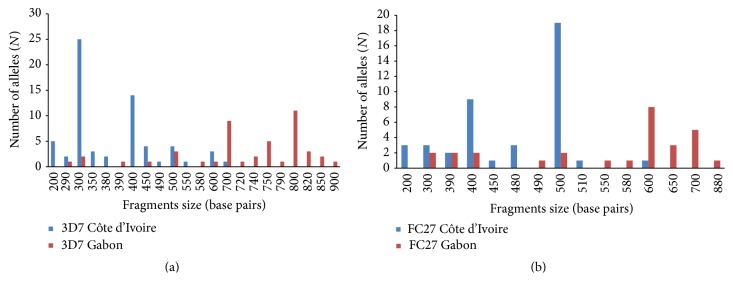
(a)* P. falciparum msp*2 3D7 type alleles classified according to the length (in base pairs) in Côte d'Ivoire and Gabon. (b)* P. falciparum msp*2 FC27 type alleles classified according to the length (in base pairs) in Côte d'Ivoire and Gabon.

Based on* msp*2 gene analysis, 12 3D7 type alleles with a size ranging from 200 to 700 bp and 9 FC27 type alleles (200–600 bp) were detected in Côte d'Ivoire. In Gabon, 16 3D7 type alleles (290–900 bp) and 11 FC27 type alleles (300–880 bp) were identified. 3D7 300 bp and FC27 500 bp alleles were the most frequent in Côte d'Ivoire while in Gabon, 3D7 800 bp and FC27 600 bp alleles were predominant (Figures 2(a) and 2(b)).

The prevalences of common alleles in the two countries were 35.7% (10/28) for* msp*1 and 38.7% (10/26) for* msp*2.

#### 3.2.3. Multiple Infections

A total of 236 (129 for* msp*1 and 107 for* msp*2) and 151 (78 for* msp*1 and 73 for* msp*2) individual* msp* fragments were, respectively, found in Côte d'Ivoire and Gabon.

The numbers of genotypes per isolate were 1 to 3 and 1 to 4 for* msp*1 gene in Côte d'Ivoire and Gabon, respectively. For* msp*2 gene, there were 1 to 5 genotypes per isolate from Côte d'Ivoire versus 1 to 4 per isolate from Gabon.

For* msp*1 gene, the overall proportion of multiple infections (MI) was 45.1% in Côte d'Ivoire versus 30.2% in Gabon (*p* = 0.083). There was the same trend with* msp*2 gene: 40.3% versus 31.4% of multiple infections in Côte d'Ivoire and Gabon, respectively (*p* = 0.312). The distributions of MI and complexity of infection (COI) according to the age of patients were shown in Table 2. Overall, the COI was 1.66 (SD = 0.79) in Côte d'Ivoire and 1.58 (SD = 0.83) in Gabon (*p* = 0.293).

## 4. Discussion

This study enabled a comparison of the genetic diversity of* P. falciparum* from infected patients living in Côte d'Ivoire versus Gabon using the most polymorphic regions of* msp*1 and* msp*2 genes. Thus, a high genetic diversity of the population of* P. falciparum* isolates from both areas was found.

Within* msp*1 gene, the high diversity is compatible with the high level of malaria transmission in both areas. Data from Gabon underline the heterogeneity of* P. falciparum* strains in this country based on genetic diversity. Indeed, a high diversity was reported in isolates from Libreville (30 alleles) during the year 2011-2012 [20] while in Franceville in the southeast only 9 alleles have been identified 12 years before [11]. It could be more accurate to conduct studies including several samples collection at different time point within the same region to assess and compare the genetic profile of parasites circulating in endemic areas [21] in an attempt to avoid intra- and interindividual variation in the number of parasite genotypes detected in the different episodes of malaria [22]. Moreover, a single blood sample may not be enough to show the whole diversity of parasites carried by an individual, since genotypes can appear and disappear in a very short time [23, 24]. The predominance of K1 allelic family in malaria endemic countries is frequently reported as found here [13, 20, 21, 25]. It has been previously shown that the RO33 family was poorly polymorphic in Gabon [20, 26], in contrast to our findings with 10 different alleles.

Considering* msp*2 allelic diversity, a predominance of 3D7 allelic family was found in both areas. This has also been reported in previous studies conducted in Benin [27], Burkina Faso [13], and Congo-Brazzaville [25]. However, the present data differ from those reported in Nigeria [28] and recently in Benin [21].

In our study, the size of* msp*1 and* msp*2 PCR products obtained from isolates collected in Gabon was longer compared to the one of the alleles detected in Côte d'Ivoire. Such difference between both areas may suggest a specific immune response against these alleles or a random event due to genetic drift [29]. However, an underestimation of such difference can be expected. One limit of the use of any marker based on DNA fragment size is convergence. In fact, similar sized fragments (but not identical) can be scored as identical leading to a false impression of similarity. Within allele families, alleles of the same size may have different amino acids motifs [10, 30]. Nevertheless, markers such as* msp*1 and* msp*2 genes are enough robust markers of polymorphism and can be used successfully to characterize genetic* P. falciparum* strains populations [13, 14].

The allelic variations as well as the presence of common alleles should be better explored by sequencing the isolates from both countries. This could be useful for designing a malaria vaccine based on study sites specificity.

For each gene, the MI was higher in Côte d'Ivoire than in Gabon but the genotypes numbers per isolate were almost equal. In Côte d'Ivoire, based on* msp*2 gene genotyping, Mara et al. [31] have found a number of clonal infections per individual, ranging from 1 to 8, with a mean of 2.88. In Gabon, 1 to 6 genotypes per isolate were reported from the* msp*1 gene analysis [20]. The number of clones coinfecting a single host can be used as an indicator of the level of malaria transmission or the level of host acquired immunity that is related to the endemicity [32–34]. During* P. falciparum* infection with several genotypes, a selection for effective transmission of sexual gametocyte stages to mosquitoes may occur and can probably be considered as a result of the presence of specific alleles which mediate the survival of the parasite inside the mosquito. Studies reported that the genetic diversity of natural populations of parasites could vary in remote sites far from each other by a few kilometers [13, 35]. Likewise, multiclonal infections may vary depending on the clinical status of the individual (asymptomatic versus symptomatic), the severity of the disease (simple cases versus severe), the malaria endemicity, the age of the patient, and the acquired immunity [13, 20, 36–38]. Although from our data further analyses are limited regarding COI, it seems that the different genotypes circulating in both areas may undergo different selective pressure leading to a variation of COI in each context. Thus, the significant decrease of the overall COI with age in Côte d'Ivoire could result in the development of premunition in highly endemic areas and the maintenance of the population's immunity through perennial exposure to mosquito bites [20]. This trend was already reported for* msp*2 gene in a rural setting of Côte d'Ivoire [39].

Considering* msp*1 gene only, it was shown that the relation between the complexity and age could change according to the site with a trend of increase in rural area and decrease in urban area [40]. In our study, we observed a significant decrease of COI with age for* msp*1 gene in Côte d'Ivoire.

In contrast, the overall COI tended to increase with age in Gabon as reported in Burkina Faso and Benin [13, 21] suggesting that younger children might still have protection from maternal antibodies or could simply mean a lower risk of multiple infections due to a lower exposure time at risk of infection [13]. This trend was significant with* msp*2 gene in our study in Gabon.

However, some authors did not find any association between the COI and age [41, 42].

## 5. Conclusion

The polymorphism in* P. falciparum *clinical isolates from both malaria endemic areas was high. The differences observed in some allelic families and in the complexity profile may suggest an impact of epidemiological facies as well as immunological response on* P. falciparum* genetic diversity.

## Figures and Tables

**Table 1:** Distribution of the different allelic families of *msp*1 and *msp*2 genes.

	Côte d'Ivoire	Gabon	*p* ^*∗*^
*msp*1	(*N*1_CI_ = 82)	(*N*1_Gab_ = 53)	

K1, *n* (%)	53 (64.6)	30 (56.6)	*0.349*
Mad20, *n* (%)	33 (40.2)	23 (43.4)	*0.716*
RO33, *n* (%)	29 (35.4)	17 (32.1)	*0.694*

*msp*2	(*N*2_CI_ = 72)	(*N*2_Gab_ = 51)	

3D7 *n* (%)	53 (73.6)	37 (72.5)	*0.896*
FC27 *n* (%)	37 (51.4)	24 (47.1)	*0.636*

^*∗*^Chi-square test.

**Table 2:** MI and COI according to the age of patients.

	Côte d'Ivoire			Gabon		
Age (years)	<5	≥5		<5	≥5	

		*msp*1				

			*p*			*p*

MI, %	70.6	38.5	0.018^*∗*^	30.8	30	0.958^*∗*^
COI (SD)	2 (0.73)	1.46 (0.72)	0.010^*∗∗*^	1.46 (0.82)	1.47 (0.80)	0.980^*∗∗*^

		*msp*2				

			*p*			*p*

MI, %	43.7	39.3	0.748^*∗*^	7.7	39.5	0.031^*∗*^
COI (SD)	1.5 (0.71)	1.48 (0.71)	0.754^*∗∗*^	1.08 (0.71)	1.55 (0.73)	0.032^*∗∗*^

		*msp*1 and *msp*2				

			*p*			*p*

MI, %	84	61.7	0.063^*∗*^	58.8	57.1	0.902^*∗*^
COI (SD)	2 (0.8)	1.58 (0.8)	0.036^*∗∗*^	1.41 (0.84)	1.63 (0.83)	0.371^*∗∗*^

^*∗*^Chi-square test. ^*∗∗*^Mann-Whitney test.

## Abbreviations

MI: Multiple infections
bp: Base pairs
COI: Complexity of infections.
